# Reduced high intensity training distance had no effect on V_La4_ but attenuated heart rate response in 2-3-year-old Standardbred horses

**DOI:** 10.1186/s13028-015-0107-1

**Published:** 2015-03-20

**Authors:** Sara Ringmark, Arne Lindholm, Ulf Hedenström, Michael Lindinger, Kristina Dahlborn, Clarence Kvart, Anna Jansson

**Affiliations:** Department of Animal Nutrition and Management, Swedish University of Agricultural Sciences, SE-75007 Uppsala, Sweden; Vetteam AB, SE-81591 Tierp, Sweden; Swedish Centre for Trotting Education, Wången, SE-83040 Alsen, Sweden; Lindenfarne Horse Park, Campbellville, Toronto, ON L0P 1B0 Canada; Department of Anatomy, Physiology and Biochemistry, Swedish University of Agricultural Sciences, SE-75007 Uppsala, Sweden

**Keywords:** Anaerobic threshold, Cardiovascular response, Hematocrit, Lactate, Heart rate

## Abstract

**Background:**

Training of Standardbred race horses aims to improve cardiovascular and metabolic functions but studies on the effects of different training strategies from breaking till racing are lacking. Sixteen horses with the goal to race as 3-year-olds were studied from breaking (1-year-olds) to December as 3-year-olds. Horses were allocated to either a control (C) or reduced (R) training program from 2 years of age. The aim was to evaluate the effect of reducing the distance of high intensity exercise by 30% with respect to velocity at lactate concentration 4 mmol/l (V_La4_), blood lactate and cardiovascular response. All training sessions were documented and heart rate (HR) was recorded. A standardized exercise test of 1,600 m was performed 10 times and a V_La4_ test was performed five times.

**Results:**

C horses initially exercised for a longer time with a HR >180 beats per minute compared to R horses (*P* < 0.05) but after 6–9 months, time with HR >180 bpm decreased in C and were similar in the two groups (*P* > 0.05). Over the 2-year period, recovery HR after the 1,600 m-test decreased in both groups but was within 2 months lower in C than in R (*P* < 0.05). C horses also had lower resting HR as 3-year-olds (*P* < 0.01) than R horses. In C, post exercise hematocrit was higher than in R (*P* < 0.05). There was a tendency (*P* < 0.1) towards a larger aortic diameter in C as 3-year-olds (C: 1.75 ± 0.05, R: 1.70 ± 0.05 cm/100 kg BW). Left ventricle diameter and blood volume (in December as 2-year-olds) did not differ between groups. There were no differences between groups in post exercise blood lactate concentration or in V_La4_. Both groups were equally successful in reaching the goal of participation in races.

**Conclusions:**

Horses subjected to a reduced distance of high intensity training from the age of 2 showed an attenuated heart rate response, but were able to maintain the same V_La4_ and race participation as horses subjected to longer training distances.

## Background

Standardbred horse race performance is positively correlated to V_La4_ [1-3] and, in general, European Standardbred horses are subjected to high intensity training for at least 1 year before the competition career can begin. Training is associated with improvements in cardiovascular responses [4], thermoregulation, aerobic and glycolytic metabolism, as well as reductions in the accumulation of extracellular potassium, reduced depletion of muscle ATP and glycogen [5]. While all physiological systems are stressed in order to improve exercise performance and quality of musculoskeletal tissues, changes within each may also contribute to fatigue during acute exercise and training [5,6] and (chronic) overload of musculoskeletal tissues. Amongst sport horses, lameness is a common cause of a defaulted race career [7,8]. Interestingly, there have been no studies comparing the effects of different training strategies applied from breaking to the start of the racing career on performance related variables in race horses. However, the volume of high intensity training is positively correlated with increased risk of musculoskeletal problems in race horses [9-11]. Accordingly, one strategy to reduce the risk of musculoskeletal overload injuries could be a reduction of the volume (distance, duration) of high intensity training.

The aim of this study was to evaluate the effect on V_La4,_ blood lactate and cardiovascular response of two training distances (control and reduced by 30%) of high intensity training over a 21-month period in 2 year old Standardbred horses with the goal to race as 3-year-olds. The control training program was designed together with group of professional trainers and estimated to be sufficient to get horses in to a condition to race by the age of 3 years.

## Methods

The study was performed at the Swedish National Trotting School, Wången, Sweden, and the horse use and testing protocols approved by the Umeå Local Ethics Committee according to the Swedish law of animal welfare.

### Horses

Sixteen Standardbred geldings born between 19^th^ of March and 15^th^ of July in 2009 were used. The horses were from four Swedish breeders, were progeny from eight sires and were chosen with the criteria to be of mainly American ancestors. The horses were taken care of and trained by high school students with supervision from professional trainers. Prior to the study, all horses had received the same training which started with breaking in September 2010 as 1-year-olds [12]. All horses had been at the national training facility for 2–4 months before the study started, and kept on the same diet and management system [12]. The study started in January 2011 as 2-year-olds and from January to the middle of March 2011 all horses were subjected to the same training program. In the middle of March 2011 horses were divided in two groups and one group was allocated to a control (C) training program and the other to a reduced (R) training program until December 2012 as 3-year-olds. The groups were balanced with respect to breeder and factors known to affect race performance in Swedish Standardbred trotters. These include sire [13] estimated genetic potential (breeding index calculated from the average of the parents [14,15], percentage of French ancestors [16], inbreeding coefficient [17], age [18,19], abnormal radiographic findings in metacarpal metatarsal and tarsal joints, conformation (subjectively determined through visual examinations: x-shaped legs, asymmetric pelvis, tied in below the knee, i.e. leg circumference smaller below knee than above fetlock), height at withers [20,21] and proportion of type IIA / type IIB muscle fibers in *musculus gluteus medius* [22] from a muscle biopsy taken in December 2010 as 1-year-olds (Table 1).

**Table 1:** **Parameters used to balance training groups**

**Group**	**Horse**	**Breeder**	**Breeding index**	**Sire**	**French, %**	**Inbreeding coefficient, %**	**Age, days**	**Height, cm**	**X-ray findings** ^**1**^	**Conformation remark**	**IIA/IIB**
**C**	1	A	106.5	1	20.6	7.2	580	153	c	0	0.73
**C**	2	A	101	1	0	15.8	653	152	d	2	1.34
**C**	3	A	107.5	1	0	13.5	535	155		0	1.58
**C**	4	A	109.5	3	26.5	7	630	157.5		1	1.27
**C**	5	B	115.5	2	0	13.5	611	156		0	1.86
**C**	6	B	114	2	10.3	8.5	600	157	b	1	1.58
**C**	7	B	106.5	4	11.0	7.7	567	153	d	2	1.50
**C**	8	C	-	5	10.2	11.1	587	154	a	1	0.83
**Mean C-group**			**108.6**		**9.8**	**10.5**	**595**	**154.7**		7*	**1.34**
**R**	9	A	107	1	0	1.8	585	155.8	c	1	1.23
**R**	10	A	-	1	15.3	9.6	612	160		0	1.29
**R**	11	A	-	1	26.5	5.7	623	150.5	a	1	1.31
**R**	12	D	113.5	6	0	12.6	569	152	a	1	0.95
**R**	13	B	113.5	2	0	9.2	631	152.5	d	0	2.35
**R**	14	B	105	2	10.3	8.4	605	161		1	1.08
**R**	15	B	115.5	7	0	14.9	565	152		0	0.98
**R**	16	C	103.5	8	0	11.5	604	158	b	1	1.56
**Mean R-group**			**109.7**		**6.5**	**10.7**	**599**	**155.2**		5*	**1.34**

^1^X-ray performed in November as 1-year-olds on tarsal, metacarpal and metatarsal joints, horses with defects validated to similar severity received the same letter and were separated between the groups. * = Total number of remarks.

Information used to allocate horses in to 2 groups (control: C and reduced: R). Groups were balanced to be as equal as possible with respect to breeder (A-D), breeding index (average of breeding index of parents, − if no breeding index for parents available), sire, part of French ancestors in pedigree, inbreeding coefficient, age (days in the 1^st^ of January as 2-year-olds), height at withers (cm), x-ray findings^1^_,_ conformation remarks (0 = no serious remark, 1–2 = remarks) and quote of percentage of type IIA / type IIB muscle fibers in *musculus gluteus medius*.

### Training

The training programs were designed together with a group of experienced professional trainers and included heat training, flat interval training and uphill interval training, which is common practice in Sweden. The training track mainly used was a 1,000 m oval banked sand track, 3 m incline that during wintertime was frozen and covered with crushed snow and ice. Throughout the study, the speed was set by the trainer and was the same in both groups (see below). Based on the correlation between post-exercise lactate response and 10-min recovery HR (y = 4,1513x + 55,944, R^2^ = 0.54) observed on these horses in March 2011, a maximal 10 min post-exercise HR of 80 bpm was aimed for (corresponding to a blood lactate concentration less than 6 mmol/L; plasma lactate ≤ 8 mmol/L; [23]) and speed was adjusted according to this in all training sessions as 2-year-olds but not as 3-year-olds. This meant that if HR10 min post exercise were > 80 bpm in an individual the speed during the next session was decreased for that individual. As there were individual variations of HR10 within training groups, two sub-training groups were created with equally many horses from each training group. These groups were exercised at a speed adjusted so that all horses should have a HR10 of max 80 bpm. The speed was also adjusted due to track conditions (muddy track, lower speed). As 3-year-olds, speed was adjusted according to the trainer’s judgement of the horses’ capability but was always the same for group C and group R.

In January 2011 as 2-year-olds, horses had recently been castrated and therefore training for 2–3 weeks was mostly walk and slow trot ~3 times/week. In February 2011, exercise in heats of 1,600 m on the oval banked race track was introduced mixed with jogging 1–2 times/week. Beginning on the 18^th^ of March 2011, all exercise sessions that were expected to cause a heart rate (HR) > 180 beats per minute (bpm) had the exercise distance reduced by approximately 30% for horses allocated to the R-group. The HR limit of 180 bpm was chosen since workloads producing a HR above that will stress both aerobic and anaerobic systems [24].

High intensity training was performed ~2 times/week. Heat exercise (C: 1–3 times 1,600 m/training session, R: 1–3 times 1,100 m/training session, 10 min walk/jog between bouts until October 2011 as 2-year-olds and therefrom 4 min walk between bouts) was performed throughout the study (Table 2). Before heat exercise horses in both groups were warmed up by jogging 3,000 m at an approximate speed of 6 m/s. The mean speed during heat exercise when horses had a HR > 180 bpm was 8.7 ± 0.1 m/s as 2-year-olds and 9.5 ± 0.1 m/s as 3-year-olds (*P <* 0.0001). After heat exercise horses were jogged 1,200 m at an approximate speed of 6 m/s before return to the stable.

**Table 2:** **Number of training sessions**

		**2-year-olds**				**3-year-olds**			
	**Group**	**January-March**	**April-June**	**July-September**	**October-December**	**January-March**	**April-June**	**July-September**	**October-December**
**Heat training**	**C**	7.0 ± 0.8	15.9 ± 2.5	11.8 ± 1.3	6.6 ± 0.6	3.6 ± 0.5	5.1 ± 1.0	10.0 ± 1.3	9.3 ± 1.3
	**R**	6.1 ± 0.8	15.6 ± 2.5	16.1 ± 1.3	8.8 ± 0.6	4.5 ± 0.5	7.0 ± 1.0	10.5 ± 1.3	6.3 ± 1.3
**Intervals, flat**	**C**			4.0 ± 0.5	6.6 ± 1.1	1.8 ± 0.1	1.8 ± 0.3	1.1 ± 0.1	0.4 ± 0.2
	**R**			5.0 ± 0.5	8.1 ± 1.1	1.9 ± 0.1	2.6 ± 0.3	1.0 ± 0.1	0.3 ± 0.2
**Uphill intervals**	**C**					6.1 ± 1.5	6.6 ± 1.3	6.3 ± 0.7	1.9 ± 0.4
	**R**					9.4 ± 1.5	8.9 ± 1.3	7.8 ± 0.7	2.1 ± 0.4
**Cross country jog**	**C**		5.5 ± 0.3	6.9 ± 0.6	3.9 ± 1.4	2.3 ± 0.5	0.3 ± 0.2	0.5 ± 0.2	
	**R**		6.0 ± 0.3	5.5 ± 0.6	4.6 ± 1.4	2.8 ± 0.4	0.25 ± 0.2	0.0 ± 0.2	
**Total training**	**C**	7.0 ± 0.8	21.4 ± 2.4	22.6 ± 1.0	17.1 ± 2.1	13.8 ± 1.7	13.8 ± 2.3	17.9 ± 1.8	11.5 ± 1.6
	**R**	6.1 ± 0.8	21.6 ± 2.4	26.6 ± 1.0	21.5 ± 2.1	18.5 ± 1.7	18.8 ± 2.3	19.3 ± 1.8	8.6 ± 1.6
**Total training**	**All**	6.6 ± 0.6	21.5 ± 1.7	24.6 ± 0.7	19.3 ± 1.4	16.1 ± 1.2	16.3 ± 1.6	18.6 ± 1.3	10.1 ± 1.1

Number of sessions performed per horse as heat training (including 1–3 times 1,600 m [C-group] or 1–3 times 1,100 m [R-group], 2,000 m tests, V_La4_–tests [4 times 1,000 m] and races [bonus race, qualification race and official races]), intervals on track (500–700 m [C: 6 times, R: 4 times]), uphill intervals (5% incline, 600 m [C: 6 times, R: 4 times]), cross country jogs and total number of training sessions including heat training, intervals on track, uphill intervals and cross country jogs for 8 horses subjected to a control training program (C) and 8 horses subjected to a reduced (by 30% distance, R) training program from March as 2-year-olds to December as 3-year-olds (lsmeans ± SE). High intensity training was scheduled ~2 times/week but horses could miss training due to health problems, lack of staff or poor weather and track conditions.

From August 2011 as 2-year-olds, interval exercise (performed at straight parts of the oval track, 500 m/interval in August and September and thereafter 700 m/interval, 1 min walk between each interval [C: 6 times 500–700 m, R: 4 times 500–700 m]) was also performed. The mean speed during interval exercise on flat ground for both groups when horses had a HR > 180 bpm were 8.3 ± 0.2 m/s as 2-year-olds and 9.3 ± 0.2 m/s as 3-year-olds (*P <* 0.001). The warm up before and jog after the intervals were the same as for heat exercise. Also, from February 2012 as 3-year-olds, uphill intervals (600 m, 5% incline, 30 m rise per interval, 4 min walk/jog downhill between each interval [C: 6 intervals, 180 m rise, R: 4 intervals, 120 m rise]) were used. Before uphill intervals horses were jogged in a hilly terrain at an approximate speed of 5 m/s for 5,500 – 6,000 m. After the last interval horses were walked 500 m back to the stable. During all interval exercise, horses from both groups were exercised together for the first 4 intervals and then C horses continued for 2 more intervals after the R horses had finished. For the uphill interval exercise when HR > 180 bpm the speed was 7.3 ± 0.9 m/s. Lactate concentrations were used to confirm workloads recommended for interval training by Lindholm and Saltin [6] (interval exercise: 5.3 ± 3.3 mmol/L after the 4^th^ interval and 7.2 ± 5.6 mmol/L after the 6^th^ interval, uphill interval exercise: 10.0 ± 1.4 mmol/L after the 4^th^ interval and 8.8 ± 1.2 mmol/L after the 6^th^ interval).

On a few occasions, as a preparation for participation in official races, heat exercise of 2,000 m was performed by all horses, since most official races in Sweden are 2,140 m.

Between training sessions both groups were also jogged on a mostly flat jog track (2-year-olds: 47 ± 8 sessions, 169 ± 175 s/session with HR > 180 bpm [n = 20], 3-year-olds: 35 ± 12 sessions 38 ± 34 s/session with HR > 180 bpm [n = 13] [mean ± SD per horse]) and at a cross country jog track (2-year-olds: 16 ± 4 sessions, 289 ± 122 s with HR > 180 bpm [n = 28], 3-year-olds: 3 ± 2 sessions, 524 ± 132 s with HR > 180 bpm [n = 26] [mean ± SD]) depending on track conditions.

Equally many training sessions were planned for horses in both groups and all training sessions (type and distance) were documented in a protocol and summarized in eight 3-month periods with the exception of the first period of 2.5 months that ended on the 17^th^ of March 2011 as 2-year-olds (Table 2). Horses that according to the head trainer’s opinion were unhealthy were left out of training for as long as he thought was needed. Training could also be cancelled due to poor weather and track conditions or lack of staff. According to a routine check list including drivers’ opinion on fitness and general condition, horses were sent for a veterinary check. After longer pauses (>3 weeks) training then started with light exercise until the horse was considered ready to join the training program of the group where it belonged. Planned periods of rest for all horses due to practical reasons for the trainer and staff occurred for one week in April and 1.5 weeks in July and October 2011 as 2-year-olds and one week in January, March, June and December 2012 as 3-year-olds.

To reflect the Swedish Standardbred racing industry, an overall goal for horses from both groups was to start competing in races at latest in the last 6 months of the 2012 year’s season as 3-year-olds. When horses were considered to be adequately trained (trainer’s opinion) they participated in a preparation race (2,140 m, velocity standardized to 10.5 - 11.4 m/s) as 2-year-olds, a qualification race (required for permission to participate in official races 2,140 m, > 11.8 m/s) as 3-year-olds and also in official races (1,600 - 2,140 m) as 3-year-olds. For the purposes of this study, these races were classified as heat exercise when calculating the amount of different types of training.

### Standardized exercise tests

Two types of standardized exercise tests were performed. The first was a single bout of 1,600 m (1,600 m-test), since the shortest race distance in Sweden is 1,640 m, with the purpose to monitor training response [25]. The test was performed 5 times when horses were 2 years old (March, May, July, August and December 2011) and 5 times when they were 3 years old (April, June, August, October and December 2012). Before each test the horses did a warm up consisting of 3,000 m of slow trot (5.6 m/s), ~ 200 m fast trot (free speed, to make sure the horse was alert), and then slow trot for 1,000 m before the test started. The test aimed for a steady speed of 10.8 m/s, with the exception of the first test which was performed in March 2011 prior to the reduced training intensity for R horses which was run at a speed of 10.0 m/s (near maximal speed that could be maintained at trot for many individuals at that time). There were no differences between groups for speed in the 1,600 m-test (10.6 ± 0.0 m/s for C and 10.7 ± 0.0 m/s for R). Within 1 min after the test was finished, a blood sample was collected in 7 mL lithium heparin-tubes^a^ and the horses jogged for 1,000 m and then returned to the stable.

The second exercise test was performed to determine the velocity at blood lactate concentration of 4 mmol/L (V_La4_) for each individual [26]. The V_La4_-test was performed 5 times as 3-year-olds (May, July, August, October and December 2012). Before the test started, horses warmed up with 3,000 m of slow trot (5.6 m/s) that finished with a ~ 200 m fast trot, followed by slow trot for another 1,000 m. The V_La4_-test comprised 4 intervals of 1,000 m with increasing speed (bout 1: 10.3 ± 0.4 m/s, bout 2: 10.7 ± 0.4 m/s, bout 3: 11.1 ± 0.4 and bout 4: 11.4 ± 0.4 m/s [mean ± SD]) with the aim of determining V_La4_ between bouts 2 and 3. Within 1 min after each bout a blood sample was collected from the jugular vein in 7 mL lithium heparin-tubes^a^. The mean ± SD blood lactate concentrations were for bout 1: 2.8 ± 1.2 mmol/l, bout 2: 3.6 ± 1.5 mmol/l, bout 3: 5.6 ± 1.9 mmol/l and for bout 4: 8.7 ± 2.0 mmol/l. Immediately after the blood sampling, the horses were jogged for 750 m, ~3 min, and then started the next bout. The tests were performed on a 1,000 m race track and the horses were tested in groups of 2–7 horses, balanced between C and R horses.

### Blood samples and analyses

Blood samples were immediately stored cooled and analyzed within 20 min for lactate with a Lactate Pro analyzer^b^, validated for use in horses at lactate concentrations ≤ 12 mmol/L [23]. Hematocrit was determined in the last blood sample in the V_La4_-test and all samples from the 1,600 m-test by centrifugation^c^ of capillary tubes for 2 minutes at 14,000 X g at ~15°C.

### Heart rate recording

Heart rate and track speeds during all training sessions and exercise tests were measured using heart rate monitors set to record at 1 Hz and synchronized with a GPS (Polar CS600X and G5 GPS sensor, Polar Electro, Finland). Data was downloaded and an analysis provided by Polar ProTrainer 5 Equine Edition Software (Polar Electro, Finland) was used. From HR recordings, only sections where HR was > 180 bpm were used and total time with HR > 180 bpm, total distance with HR > 180 bpm and average speed when HR > 180 bpm were calculated. Also, HR10 min after the intensive exercise and the highest recorded HR for each horse and period was determined from the HR curves. Curves with missing values of more than 20 s in the span > 180 bpm were discarded. After removal of records of poor quality, 1,093 curves remained.

Data were categorized by the type of exercise; heat training, interval training on flat ground and uphill interval training. After the 1,600 m-test an average HR for the 10^th^ min after passing the finish line was calculated from the HR-meter output (HR10). Heart rate at 10 min was chosen because of the very rapid decline in HR within 10 min after exercise and for practical reasons, i.e. horses had returned to the stable and HR measurements could be made indoors, while standing still in a calm environment. HR at V_La4_ was estimated from the last 30 s of each 1,000 m bout in the V_La4_-tests and calculated using exponential regression.

Resting HR was recorded by 2 different methods in January as 2- and 3-year-olds and in December as 3-year-olds. In method 1, HR was determined by auscultation with a phonendoscope in the evening with no other activities in the stable, thus securing a calm and well known environment for the horses. In method 2, horses were fitted with HR meters (Polar CS600X) and HR recorded during night time when quiet. From the HR-meter output the lowest mean of at least 10 min of a consecutive recording were used as resting HR.

### Cardiovascular assessments and plasma aldosterone

When horses were at rest, measurement of blood volume were performed in December 2011as 2-year-olds using Evans blue dye dilution as described previously [27] and a hematocrit value obtained from the last V_La4_ sample (V_La4_ test performed the same day). Echocardiograms were recorded using ultrasound^d^ and a 1.7-3 mHz probe in January as 2-year-olds, December as 2-year-olds and December as 3-year-olds. Left Ventricular Internal Diameter during diastole (LVIDd) and aorta diameter were recorded and calculated as recommended by Pattesson [28].

Respiratory rate (RR) was recorded at the same time as resting HR by auscultation with a phonendoscope and visual inspection. Blood pressure (BP) was measured at rest with high definition oscillometric (HDO) technique (S + B med VET GmbH, Germany) with a cuff placed at the root of the tail.

Plasma aldosterone concentration was analysed to assess sodium status which could affect cardiovascular responses. The concentration was determined after extraction of fat and proteins (acetone and petroleum ether extraction) by use of a radioimmunoassay kit (Coat-a-Count, DPC, Los Angeles, CA), earlier used in horses [29,30]. The samples for the standard curve were extracted in the same way. The quality control was run using MultiCalc software version 2.0 (Wallac, Turku, Finland). The within and between assay variation was < 10%. Recovery in diluted samples was 95.3%. There was no difference in plasma aldosterone concentration between groups (C-group: 244 ± 37 pmol/L and R-group: 264 ± 37 pmol/L, *P >* 0.05).

### Feeding and management

Horses were fed grass haylage *ad libitum* and had been adapted to *ad lib* feeding for 4 months prior to the study. Average body condition score according to Henneke *et al*. [31] (scale 1–9) were maintained around 5 for the whole study period. From the end of April until October some grass pasture was available in the paddock. The haylage used mainly consisted of Meadow fescue, Timothy and Ryegrass. In total 5 batches were used during the study (47–71% DM content, 10.3-11.7 MJ ME and 93–164 g CP per kg of DM), both 1^st^ and 2^nd^ cuts. The diet was complemented with 0.25 - 1 kg (depending on haylage nutrient content) of a pelleted Lucerne product^e^ to enhance mineral supplement intake and meet the CP requirements [32]. A commercial mineral supplement^f^ was used to meet vitamin and mineral requirements except for between September 2011 as 2-year-olds and May 2012 as 3-year-olds when the only supplement used was a selenium and Vitamin E product^g^ due to sufficient content of Ca, P and Mg in haylage during this period. Sodium chloride was always provided from salt blocks placed in a feed box within each stall and from September year as 2-year-olds an additional 15 g of NaCl was fed together with lucerne and other minerals due to expected increased sweat losses. Water was offered from two 20 L buckets in the stall that were refilled twice a day, and also provided from a big tub in the outdoor paddock.

Horses were stabled individually in 9 m^2^ boxes for approximately 8 hours / day and spent the rest of the time in an outdoor paddock (approximately 20 000 m^2^) with access to shelters. Wood shavings were used as litter in boxes and shelters. Hoof trimming and shoeing was performed every 5 - 6^th^ week, and during wintertime (October/November-March) ice chalks were mounted to the shoes permanently.

### Calculations and statistical analyses

All statistical analyses were performed using Statistical Analysis Systems package 9.3 (SAS Institute Inc., Cary, NC, USA). From HR-recordings of training sessions, time, distance and speed where HR > 180 bpm, HR10 min post exercise and maximum HR were analyzed for differences between groups (control or reduced), periods (1–8 as described in the training section) and interaction of group and period using a mixed model (proc mixed). We also modelled correlations between repeated measurements of individuals using the model:

$$ {\mathrm{Y}}_{\mathrm{i}\ \mathrm{j}\ \mathrm{k}} = \upmu + {\mathrm{a}}_{\mathrm{i}} + {\upbeta}_{\mathrm{j}} + {\upgamma}_{\mathrm{k}} + {\left(\upbeta \upgamma \right)}_{\mathrm{j}\mathrm{k}} + {\mathrm{e}}_{\mathrm{i}\ \mathrm{j}\ \mathrm{k}} $$

where Y_i j k_ is the observation, μ the mean value, a_i_ the effect of individual, β_j_ the effect of group, γ_k_ the effect of period, and e_i j k_ the residuals with a spatial power correlation structure between periods (SP-POW).

The number of training sessions performed by each horse was also analyzed in a mixed model for the same effects using an unstructured covariance structure (UN):

$$ {\mathrm{Y}}_{\mathrm{i}\ \mathrm{j}\ \mathrm{k}} = \upmu + {\mathrm{a}}_{\mathrm{i}} + {\upbeta}_{\mathrm{j}} + {\upgamma}_{\mathrm{k}} + {\left(\upbeta \upgamma \right)}_{\mathrm{j}\mathrm{k}} + {\mathrm{e}}_{\mathrm{i}\ \mathrm{j}\ \mathrm{k}} $$

where Y_i j k_ is the observation, μ the mean value, a_i_ the effect of individual, β_j_ the effect of group, γ_k_ the effect of period, and e_i j k_ the residuals where all covariance’s between periods are estimated separately.

To assess the effect of training status on exercise test variables, the number of training sessions that each individual performed during the last 3 weeks before each exercise test was summarized and classified as 1: 0–2 training sessions, 2: 3–5 training sessions and 3: ≥ 6 training sessions. Training status was then included as a fixed effect in the models for V_La4_ and 1,600 m-test. From the V_La4_-test, blood lactate concentration (mmol/L) was plotted against speed (m/s) and exponential regression analyses were used to estimate individual V_La4_ (MS Office Excel 2010). V_La4_ and hematocrit from the last blood sample in the V_La4_-test, and blood lactate, HR10 and hematocrit from the 1,600 m-tests were analyzed for differences between groups and occasions when the test was performed by the following model:

$$ {\mathrm{Y}}_{\mathrm{i}\ \mathrm{j}\ \mathrm{k}\ \mathrm{l}\ \mathrm{m}} = \upmu + {\mathrm{a}}_{\mathrm{i}} + {\upbeta}_{\mathrm{j}} + {\upgamma}_{\mathrm{k}} + {\left(\upbeta \upgamma \right)}_{\mathrm{j}\mathrm{k}} + {\updelta}_{\mathrm{l}} + {\upeta}_{\mathrm{m}} + {\mathrm{e}}_{\mathrm{i}\ \mathrm{j}\ \mathrm{k}\ \mathrm{l}\ \mathrm{m}} $$

where Y_i j k l m_ is the observation, μ the mean value, a_i_ the effect of individual, β_j_ the effect of group, γ_k_ the effect of occasion, δ_l_ the effect of speed (1,600 m-test only) η_m_ the effect of training status and e_i j k l m_ the residuals following an ARH(1), i.e. an autoregressive structure with heterogeneous variances, for the different occasions.

Resting HR, RR and BP were analyzed for differences between groups using the first value as in January 2011 as 2-year-olds as a covariate in the following model:

$$ {\mathrm{Y}}_{\mathrm{i}\ \mathrm{j}\ \mathrm{k}\ \mathrm{l}} = \upmu + {\mathrm{a}}_{\mathrm{i}} + {\upbeta}_{\mathrm{j}} + {\upgamma}_{\mathrm{k}} + {\left(\upbeta \upgamma \right)}_{\mathrm{j}\mathrm{k}} + {\updelta}_{\mathrm{l}} + {\mathrm{e}}_{\mathrm{i}\ \mathrm{j}\ \mathrm{k}\ \mathrm{l}} $$

where Y_i j k l_ is the observation, μ the mean value, a_i_ the effect of individual, β_j_ the effect of group, γ_k_ the effect of age, δ_l_ the effect of starting value as 1-year-olds and e_i j k l_ the residuals following an ARH(1).

For all the above analyses, if the overall effect of interaction between group and occasion was *P* < 0.1, a separate analysis with this excluded was run including the effects of age and group alone. If no overall effect of interaction was present, *P* values for effect of group is reported from this analysis. For differences between periods for all horses, data were also analyzed with the effect of group excluded.

Differences between groups in plasma volume, aldosterone and mean HR at V_La4_ for 13 horses (due to missing values in 3) were analysed using GLM procedure. Also, a Pearson’s correlations test was performed where all horses were treated as one group to study possible correlations between lactate, HR10 and hematocrit for the 1,600 m-tests.

Differences were considered as significant if *P <* 0.05. Values are presented as least squared means ± standard errors, *P* values in text are from ANOVA tables if nothing else is declared.

## Results

### Training with HR > 180 bpm

**Figure 1:**
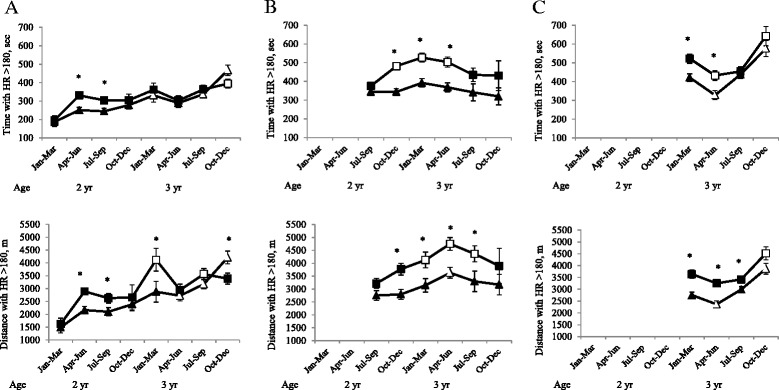
**High intensity training time and distance.** Mean time and distance per training session with heart rate (HR) > 180 bpm in 16 horses subjected either to a control training program (squares) or a reduced (by 30% distance) training program (triangles) from the middle of March as 2-year-olds until December as 3-year-olds. Training was performed as heat training **(A)**, interval training on flat track **(B)** or uphill interval training **(C)**. * Indicates a difference (*P* < 0.05) between the control and reduced group. Unfilled dots indicate a significant difference within group from **(A)** January-June as 2-year-olds, **(B)** from July-September as 2-year-olds and **(C)** from January-March as 3-year-olds.

As expected, C horses initially exercised for a longer distance with a HR > 180 bpm in heat exercise (*P <* 0.05), flat ground interval exercise (*P <* 0.0001) and uphill interval exercise (*P <* 0.0001) but after 6–9 months, time with HR > 180 bpm decreased in C and was no longer significantly different from R (*P* > 0.05). Overall, the C horses exercised for a longer time (Figure 1) with HR > 180 bpm in the interval training sessions (flat; *P <* 0.001 and uphill; *P <* 0.01) but there were no overall differences between groups in the heat exercise or cross country jogs (*P* > 0.05). However, for heat exercise there was an interaction of group and period where C horses exercised with a HR > 180 bpm for a longer time in period 2 and 3 (19^th^ of March-end of September 2011as 2-year-olds) than R. The speed when HR >180 bpm was the same for both groups during interval exercise, but during heat exercise C horses had a higher speed (9.2 ± 0.1 m/s vs. 8.9 ± 0.1 m/s, *P <* 0.001).

The highest recorded HR did not differ between groups (225 ± 2 bpm vs. 227 ± 2 bpm for C and R, respectively, *P >* 0.05), and peak HRs were lower in the first 3 months than in the last 18 months (216 ± 3 and 227–231 ± 3 bpm, respectively, *P <* 0.01). In heat exercise, highest recorded HR were in C 213 ± 1 bpm as 2-year-olds and 223 ± 1 bpm as 3-year-olds and in R 216 ± 1 bpm as 2-year-olds and 223 ± 1 bpm as 3-year-olds. In the interval exercise highest recorded HR were 216 ± 2 bpm in C and 219 ± 1 bpm in R and in the uphill interval exercise it was 221 ± 1 bpm in C and 224 ± 1 bpm in R, respectively.

### Lactate response and hematocrit

**Figure 2:**
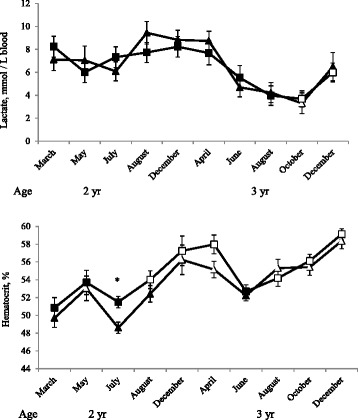
**Lactate and hematocrit response.** Lactate (mmol/L blood) and hematocrit (%) within 1 minute after a 1,600 m-test at an oval racetrack in 16 horses divided in a control (squares) and a reduced (by 30% distance, triangles) training program since March as 2-year-olds. * Indicate difference (*P* < 0.05) between the control and reduced group. Unfilled dots indicate a significant difference from the starting point in March as 2-year-olds within group. Number of participating horses in each test: 12, 8, 15, 11, 15, 12, 11, 13, 13, 12.

There were no effects of group, interaction of group and occasion or training status (number of training sessions 3 weeks prior to test) on blood lactate concentration following the 1,600 m test (Figure 2). However, there was a significant effect of occasion and lactate was lower in October 2012 as 3-year-olds for both groups and also in December 2012 for C but not R compared with the first test in March 2011 as 2-year-olds. There was no difference in V_La4_ between groups and the highest V_La4_ was observed in July to August 2012 (Table 3).

**Table 3:** **Velocity at blood lactate concentration 4 mmol/l (V**_**La4**_ **) and haematocrit in V**_**La4**_ **-tests**

**Month as 3-year-olds**	**N**	**V**_**La4**_ **(m/s)**	**Hematocrit (%)**
**May**	14	10.7 ± 0.1^a^	54 ± 1^a^
**July**	10	11.0 ± 0.1^b^	55 ± 1^a^
**August**	11	11.0 ± 0.1^b^	56 ± 0^a^
**October**	10	10.8 ± 0.1^ab^	57 ± 1^b^
**December**	9	10.6 ± 0.1^a^	55 ± 1^a^
***P*** **-value**		0.0215	0.2504

^a, b^Means within a column with different superscript letters differ (*P* < 0.05).

V_La4_ (m/s) and hematocrit (%) after four intervals of 1000 m in 16 3-year-old horses trained in either a control (C-group) or reduced (by 30% distance, R-group) training program since March as 2-year-olds (lsmeans ± SE). N = number of horses participating in each test. There were no differences between groups within occasions.

The hematocrit increased during the study (Figure 2 and Table 3) and was higher overall in C than in R both after the V_La4_-test (56 ± 0 and 55 ± 1, respectively, *P <* 0.05) and after the 1,600 m-test (Figure 2, *P <* 0.05). In the 1,600 m-test there was also an effect of occasion where haematocrit in all horses increased from 50 ± 1% in March 2011 as 2-year-olds to 59 ± 1% in December 2012 as 3-year-olds but there was no interaction between group and occasion and no effect of training status in either of the tests (Figure 2 and Table 3).

### Recovery heart rate, resting HR and HR at V_La4_

**Figure 3:**
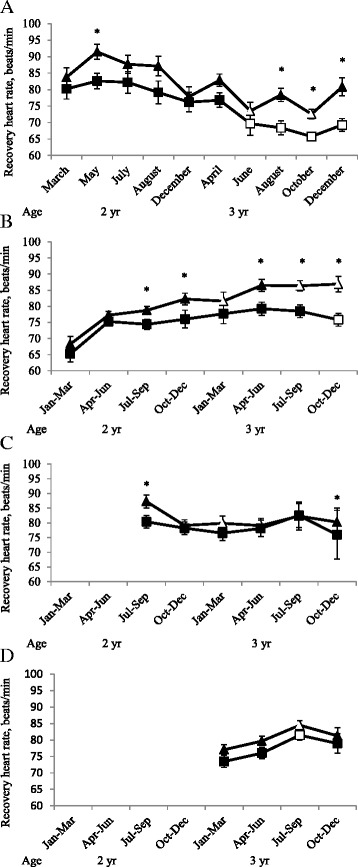
**Recovery heart rate.** Heart rate 10 min after finishing 1,600 m-tests **(A)**, number of participating horses in each test: 12, 8, 15, 11, 15, 12, 11, 13, 13, 12,), after training sessions performed as heat training **(B)**, interval training on flat track **(C)** and uphill interval training **(D)** in 16 horses divided in a control (squares) and a reduced (by 30% distance, triangles) training group since March as 2-year-olds * Indicate difference (*P* < 0.05) between the control and reduced group. Unfilled dots indicate a significant difference within group from **(A)** the starting point in March as 2-year-olds, **(B)** from January-June as 2-year-olds, **(C)** from July-September as 2-year-olds and **(D)** from January-March as 3-year-olds.

The HR10 following the 1,600 m-test was lower in C than in R (*P <* 0.0001) and in August, October and December 2012 as 3-year-olds compared with the starting value in March 2011 as 2-year-olds (Figure 3). For both groups, HR10 following the 1,600 m-test, compared to values in March as 2-year-olds, was lower in October as 3-year-olds and in December as 3-year-olds for C only. The HR10 after uphill interval exercise was higher for R (*P <* 0.05). The HR10 after heat exercise was higher in R (P < 0.0001) with significant differences for all periods except 1, 2 and 5. In the 1,600 m-test, HR10 was positively correlated with blood lactate concentration (*r* = 0.28, *P <* 0.05) and negatively correlated with hematocrit (*r* = − 0.41, *P <* 0.0001). There were no effects of training status on HR10 following any of the exercise regimens.

The resting HR was higher in R than in C, both overall and as 3-year-olds (*P <* 0.05) when both method 1 (auscultation) and method 2 (HR-meter) were used. In both groups, resting HR decreased from 1-year-olds to 2- and 3-year-olds when measured with method 1 but only in C when measured with method 2 (Table 4). There was no difference in HR at V_La4_ between groups (C: 211 ± 3 bpm; R: 217 ± 3, *P* > 0.05).

**Table 4:** **Left ventricle- and aortic diameter, blood pressure and resting heart rate**

**Age**	**January 2 year**		**December 2 year**		**December 3 year**	
**Group**	**C**	**R**	**C**	**R**	**C**	**R**
**N**	**8**	**8**	**8**	**8**	**8**	**7**
**LVIDd** ^**1**^	2.34 ± 0.09	2.33 ± 0.09			2.40 ± 0.09	2.28 ± 0.09
**Aortic diameter** ^**1**^	1.70 ± 0.05	1.75 ± 0.05			1.75 ± 0.05	1.70 ± 0.05
**RR** ^**2**^	11 ± 1	12 ± 1^a^	9 ± 0	9 ± 0^b^	10 ± 1	11 ± 1^b^
**Systolic BP** ^**3**^	116 ± 4	117 ± 4	116 ± 5	114 ± 5	120 ± 4	109 ± 5
**Diastolic BP** ^**3**^	72 ± 3	73 ± 3	64 ± 3	67 ± 3	66 ± 4	67 ± 4
**Mean arterial BP** ^**3**^	87 ± 3	89 ± 3	83 ± 4	84 ± 4	86 ± 4	82 ± 4
**Resting HR1** ^**4**^	47 ± 2^A^	50 ± 2^a^	41 ± 2^B^	41 ± 2^b^	35 ± 1^B^	41 ± 1^b^*
**Resting HR2** ^**4**^	39 ± 1^A^	38 ± 1^a^	36 ± 1^B^	39 ± 1^a^	36 ± 1^B^	40 ± 1^a^*

^1^Cm/100 kg BW.

^2^Breaths per minute.

^3^mmHg.

^4^beats per minute.

*indicates a difference between groups within age (*P <* 0.05).

^a,A^Different superscript letters indicates a difference (*P <* 0.05) within group between occasions, upper case letters = difference in C-group, lower case letters = difference in R-group.

Left ventricle inner diameter diastole (LVIDd) and aortic diameter in 16 1- and 3-year-old Standardbred horses allocated to either a control (C) or a reduced (R) (by 30% distance) training program and resting heart rate determined with phonendoscope (Resting HR1) and HR-meters (Resting HR2), respiratory rate (RR), systolic blood pressure (BP), diastolic BP and mean arterial pressure at 1, 2 and 3 years of age in the same horses (lsmeans ± SE).

### Blood volume, echocardiogram and blood pressure

Blood volume was similar in both groups (C: 55.9 ± 1.5 L and R: 54.8 ± 1.5 L, *P >* 0.05).

There was a tendency (*P <* 0.1) for an interaction of group and age for diameter of aorta /100 kg BW but no overall effect of group or age (Table 4). The left ventricular internal diameter during diastole (LVIDd) was not affected by group or age. There was no difference in RR between groups, but in R the RR was lower as 2-year-olds than as 1-year-olds (Table 4). There were no differences between groups in systolic BP, diastolic BP or mean arterial pressure (Table 4). When all horses were analyzed as one group, the RR and diastolic BP decreased from the age of 1 year until the age of 2 years (11 ± 1 and 9 ± 0 breaths/min and 72 ± 2 and 66 ± 2 mmHg, respectively).

## Discussion

### General

This study shows that reducing the training distance by 30% did not affect muscle aerobic capacity, assessed as V_La4_, by the time horses were expected to race as 3-year-olds. The training programs used were also efficient in terms of getting horses fit for race as horses from both groups were able to pass a national qualification race and preparation race to a higher extent than the rest of the cohort of 2009 in training. Experimental horses also participated in true races as 3-year-olds to the same extent as the rest of the cohort [15]. However, the C horses showed, already within the first 2–8 months of training, more pronounced cardiovascular training responses indicated by the increased hematocrit and lower HR during and after exercise. These cardiovascular differences appear however, not to have any major impact on the goal to be fit for racing which is in accordance with earlier observations that V_La4_ has a stronger correlation to race performance than HR during exercise [3].

### Effects on circulation

Amongst the tested cardiovascular variables, recovery HR responded most rapidly to training distance and was within 2 months lower in C than in R. However, a significant decrease within groups was not observed until June 2012 as 3-year-olds and by the end of the study, both groups showed an improvement (reduction) of 17–19 beats (~20%) following the 1,600 m-tests. The rapid cardiovascular responses observed, especially in C, were consistent with earlier studies. Sixteen weeks of training may increase maximal oxygen consumption by as much as 19% [33]. Part of the improvement might be due to the increased hematocrit where a 10% increase was associated with a 5% higher circulating O_2_ volume [34]. Possible reasons for a decreased HR include increased stroke volume [35] and cardiac output [36], increased plasma volume [37], increased capillary density of skeletal muscles [38] and increased muscle oxidative capacity with increased fiber type IIA/IIB ratio [39]. In the present study there was no support for an increased cardiac volume based on the size of the left ventricle, nor of blood volume. This is in contrast to Buhl and Ersbøll [40] where an increase in the size of the left ventricle with training and age was reported for Standardbred horses at the age of 2–3.5 years. The reason for lack of response in the size of the left ventricle in the present study is unclear but may be due to a low number of observations. However, the aortic diameter showed a tendency to be larger in C compared to R indicating increased central circulatory conductance. Reports of aortic adaptations to training in horses are scarce but a larger aortic dimension has earlier been observed in human endurance athletes compared to strength training athletes [41].

There was also a more rapid adaptation of cardiovascular response in C than R with respect to HR during exercise. Within 6–9 months of training the time spent at HR > 180 bpm did not differ between groups, despite the longer exercise distance in C. With heat training, the C horses trotted at a higher speed than the R when HR > 180 bpm, which shows the greater aerobic capacity in C, than R, horses. This agrees with previous studies showing that HR during exercise may be reduced by 10–20 bpm in horses subjected to 4–10 weeks of training with HR 150 bpm for 20–30 min [42] or 4–10 min of interval training [43]. The comparatively fast and marked effects on HR during exercise observed in these studies might be due to a low initial level of fitness, since the rate of improvement seems to decline the more fit the horse gets [26,44]. Evans and Rose [36] showed no change in exercise HR response during 7 weeks of training at mean velocities of 4–8.3 m/s and Lindner *et al*. [45] did not find any improvement in the velocity at HR 180 bpm in horses trained at a blood lactate concentration of 10 mmol/L for 6 weeks, in detrained and untrained horses. In the present study, horses had been in training for 6 months prior to the first test and a more marked HR reduction could probably not be expected. The present study also showed a clear effect of training on HR at rest. A reduction in HR at rest with increased fitness is a well-known effect in human athletes [46] but the same response has not until very recently [47] been documented in horses and never in horses at the same age submitted to different levels of training. For measurement of resting HR the use of HR meters during night time can be recommended since excitement, especially when horses were 1 year old, probably affected the manual measurements with a stethoscope.

There was no effect of training on BP and RR which agrees with earlier observations on horses subjected to shorter training periods of 10–16 weeks [48,49].

### Effects on lactate response

The ability to achieve a similar V_La4_ with both training volumes demonstrates that the rapidity of skeletal muscle submaximal metabolic adaptation (i.e. oxidative capacity) was not impaired by reduced distance of high intensity training. This implies that the volume (duration) of the training sessions may not be a crucial point in improving V_La4_ once volume is above an as yet undetermined threshold. Once above the volume threshold, intensity (speed or load; i.e. total muscle fibre recruitment) or repetitions (number of training sessions [50]) are likely of greater importance.

When training as 3-year-olds, there was an increase in V_La4_ in all horses during the summer and autumn period compared to the first test performed in the spring but V_La4_ then decreased in the last test performed in December. In the same month, higher lactate concentration, hematocrit and recovery HR was observed with the 1,600 m-test. These effects are probably attributed to additional stress associated with poorer training conditions during winter (altered track surface, heavier shoes on horses and clothing of drivers increasing weight and air friction for example), and possibly other reasons such as impaired health status. In general, field tests have been shown to have a good reproducibility in French Standardbred trotters when performed on the same track [51], such as occurred in the present study. However, differences in the physiological responses to different tracks have been reported [52] which may be comparable to seasonal changes in track conditions in the present study. By testing equally many horses from each group at the same time (all horses exercised in group), the risk of unbalanced conditions were minimized.

### Assessment of training progress

This study confirms that both working and recovery HR are valuable measurements to detect training progress. Recovery HR is easy to measure with a stethoscope and can therefore be used by trainers as part of their routine to follow the development of a horse. Measuring recovery HR may also provide a more reliable measurement than submaximal heart rate as HR-meters may lose contact for periods during exercise. In contrast, measurement of lactate in response to our 1,600 m-test appears to be of limited value to assess short-term training effects of young Standardbred horses since the concentration did not decrease significantly until October 2012 as 3-year-olds. This is similar to observations by Ronéus *et al*. [25] who measured lactate responses to a submaximal work in Standardbred trotters at 24, 26, 29 and 40 months of age where a significant decrease was observed only at 40 months. It shall also be noted that the post-exercise sample may not represent the maximal blood lactate response to the test since peak plasma lactate concentration may occur 0–10 min post exercise [53]. To test V_La4_ as in the present study appears to be too imprecise to indicate training response.

Interestingly, lost training three weeks prior to exercise tests did not affect metabolic and cardiovascular responses significantly since there were no effects of training status (quantity of training three weeks prior to the tests) on the HR and lactate responses. Earlier studies on the effect of reduced activity on oxygen uptake, HR and lactate responses to exercise are contradictory [43,54-57]. The variation in responses between studies might be due to the level of fitness in the horses, to the volume of reduction and to a low number of observations in each study.

## Conclusions

Horses subjected to a reduced distance of high intensity training from the age of two showed an attenuated training effect on the cardiovascular system, but were able to maintain the same muscle metabolic system responses to submaximal exercise and race participation as horses subjected to a longer training distance. This implies that the duration of training sessions, at least within the interval used in the present study, may not be a crucial point for achieving race fitness and future studies need to investigate the importance of exercise intensity and number and frequency of training sessions. As is now common practice, measurement of recovery HR after a standardized test is recommended to monitor training progress.

### Endnotes

^a^Venosafe, Terumo Europe, Leuven, Belgium.

^b^Arkray Factory Inc., Koji Konan-cho, Koka, Shiga, Japan.

^c^Hemokrit 4, Lic Instruments, Stockholm, Sweden.

^d^Vivid 3, GE Healthcare, General Electrics, UK.

^e^95% Lucerne, 5% molasses, Krafft AB, Sweden.

^f^50-150 g of Miner Röd, content/kg: Ca, 110 g; P, 17 g; Mg, 60 g; NaCl, 125 g; Cu, 1 200 mg; Se, 15 mg; vitamin A, 200,000 IU; vitamin D_3_, 10,000 IU; and vitamin E, 15,000 mg or 150 g of Miner Vit content/kg: Ca 55 g; P 65 g; Mg 60 g; NaCl 125 g; Cu 900 mg; Se 15 mg; vitamin A 100,000 IU; vitamin D_3_ 10,000 IU; and vitamin E 5,000 mg, Krafft AB, Sweden.

^g^Protect E-Selen, Lantmännen Lantbruk, Sweden.

## Abbreviations

BP: Blood pressure
bpm: Beats per minute
C: Group of horses submitted to a control training program
HR: Heart rate
LVIDd: Left ventricle internal diameter at diastole
R: Group of horses submitted to a reduced distance training program
Resting HR1: HR determined by phonendoscope
Resting HR2: HR determined from HR-meters
RR: Respiratory rate
V_La4_: Velocity at blood lactate concentration of 4 mmol/litre
